# Unveiling the influences of prenatal and maternal factors on the journey of an autistic child

**DOI:** 10.3389/fpsyt.2024.1467821

**Published:** 2024-12-20

**Authors:** Ahmed Abdelkader, Faten AlRadini, Ashwaq Alosaimi, Abdallah Abbas, Zaki Judeh, Tahneed Emy Abu Esaid, Alaa Saleh, Jaffer Shah, Samar Amer

**Affiliations:** ^1^ Faculty of Medicine, New Vision University, Internship at Beni Suef University Hospital, Faiyum, Egypt; ^2^ Department of Family and Community medicine, College of Medicine, Princess Nourah bint Abdulrahman University, Riyadh, Saudi Arabia; ^3^ Senior Nursing Specialist, General Administration of Health Programs and Chronic Diseases in Mother’s Health, Ministry of Health, Riyadh, Saudi Arabia; ^4^ Faculty of Medicine, Al-Azhar University, Damietta, Egypt; ^5^ Faculty of Medicine, New Vision University, Internship at Psychiatric hospital Beersheva, Beer Sheva, Palestine; ^6^ Faculty of Medicine, New Vision University, Nazareth, Georgia; ^7^ Faculty of Medicine, New Vision University, Chtoura, Lebanon; ^8^ Medical Research Center Department, Kateb University, Kabul, Afghanistan; ^9^ Department of Public Health and Community Medicine, Faculty of Medicine, Zagazig University, Zagazig, Egypt; ^10^ General Administration of Health Programs and Chronic Diseases in Mother’s Health, Ministry of Health, Riyadh, Saudi Arabia

**Keywords:** autism spectrum disorder, Saudi Arabia, antenatal, natal, prenatal, pregnancy, The LIVES Daily Hassles, maternal lifestyle factors

## Abstract

**Background:**

Autism Spectrum Disorder (ASD) is a complex lifelong neurodevelopmental disorder with a high and increasing global prevalence. Although the precise causes are unknown, both genetic and environmental factors, including maternal ones during pregnancy, significantly influence its development. Therefore, this study endeavors to explore the potential causes of autism, including maternal and paternal prenatal risk factors, as well as antenatal and natal maternal risk factors, and their associations with the severity of ASD in mothers of children with ASD, from February to May 2024.

**Methods:**

At an autism center in Saudi Arabia, this cross-sectional study enrolled 168 mothers of children diagnosed with ASD. The web-based survey employs a structured questionnaire to gather comprehensive prenatal, natal, and demographic data. The collected data was coded and analyzed using suitable tests.

**Results:**

The majority of the surveyed 168 mothers with autistic children reported having autism spectrum disorder (43.8%), moderate autism (31.9%), mild autism (15.6%), and severe autism (8.8%). Most autistic children had a history of one or both maternal and/or paternal antenatal exposures: 79.2% had soft drink consumption, 35.1% smoked, 24.4% had chronic physical diseases, and 20.8% had psychological disease. *Regarding maternal antenatal conditions*, 37% had a history of recurrent infection, 29.2% had anemia, 15.5% had a history of threatened abortion or bleeding, as well as exposure to air pollution, and 22 (13.1%) had a history of gestational diabetes. Significant (*p <*0.05) predictors of severe autism were gestational diabetes aOR 4.553 (95% CI: [1.518, 14.25], birth oxygen desaturation 4.142 (95% CI: [1.437, 12.45]. Furthermore, the likelihood of classifying a child’s ASD as severe increases by 7.1% with each year of age1.071 (95% CI: [1.002, 1.15].

**Conclusion:**

ASD is a prevalent health condition that has many interrelationships with prenatal, maternal (medical, environmental, and psychosocial factors), and natal conditions. Prospective studies are essential for understanding and addressing these ASD risk factors.

## 1 Introduction

Autism spectrum disorder (ASD) is a complex neurodevelopmental disorder characterized by difficulties in social communication, interaction, and behavioral patterns that can affect everyone, manifest typically in early childhood, and have a significant impact on both individuals and society (1, 2). Researchers have identified approximately 1 in 68 children with some form of ASD. Despite the high global prevalence of ASD, which has increased over the past 50 years from 1:10000 live birth to 1:59 live birth, it remains incurable and, like any other childhood disorder, requires treatment and intervention. Although the precise causes are unknown, both genetic and environmental factors, including maternal factors during pregnancy, significantly influence its development (3, 4).

Many maternal factors can impact a child’s health in several ways. The uterine environment can be changed by a number of things, including polycystic ovary syndrome (PCOs), prenatal hyperandrogenism (male sex hormones), chronic anovulation, insulin resistance, metabolic syndrome, and chronic low-grade inflammation. These things may play a part in neurodevelopmental disorders like ASD, but the exact ways they cause these disorders are still not fully understood (5–7). Using some drugs (antidepressants, specifically selective serotonin reuptake inhibitors) during the second or third trimester of pregnancy may increase the risk of a child developing ASD (8).

Several studies have indicated that mothers with gestational diabetes mellitus (GDM)-associated altered glucose metabolism and insulin resistance conditions lead to neurodevelopmental disorders such as ASD. Moreover, pregnancy-associated inflammatory processes and oxidative stress may contribute to these adverse outcomes (9, 10). Additionally, tobacco smoke significantly impacts sperm DNA methylation and gene expression, potentially altering neuronal structure and leading to adverse birth outcomes such as low birth weight and limited growth in the fetus (11). Proper maternal nutrition during pregnancy plays a crucial role for optimal fetal development, growth, and health (12).

An autistic child prefers playing alone, avoids eye contact, has limited or repetitive patterns, and is at higher risk for injuries or abuse. This can sometimes be harmful. These deficits make it difficult for many with ASD to live independently. Therefore, family members and care providers deal with emotional, financial, and even physical stress and sexual health issues while raising a child with ASD, which appears to contribute to a general decrease in parental well-being and an increase in mental health concerns. The mother of an autistic child faces more stress, anxiety, depressive symptoms, social withdrawal, and fear compared to other mothers. Similar stages of grief, such as denial, anger, bargaining, depression, and acceptance, were experienced by others, along with a feeling of guilt as if they were responsible (13–16).

ASD research is scarce in developed nations like Saudi Arabia; hence, most clinical procedures are based on Western findings (17). Thus, the above-mentioned factor heightened interest in studying this topic, which is understandable. From February to May 2024, we conducted this cross-sectional study to investigate the severity level of ASD, the prenatal, maternal, and natal risk factors that might be associated with it during pregnancy, and their interrelationships among the mothers of ASD children.

## 2 Methods

### 2.1 Study design and setting

From February 2024 to May 2024, we conducted this web-based cross-sectional study in the Society of Autism Families in SA’s large cities, Riyadh, Jeddah, and Dammam. Despite SA’s vast size, most of its services are only available in its largest cities.

### 2.2 Participants

This cross-sectional study specifically targeted adult mothers who had received an ASD diagnosis for their children. Participants were eligible if their children were less than 18 years old, attended a pediatric outpatient clinic or an autism-specific center, and met the ASD diagnostic criteria. The American Psychiatric Association’s Diagnostic and Statistical Manual, Fifth Edition (DSM-5) provides information to help diagnose ASD. According to DSM-5 (16), a child must have persistent deficits in each of three areas of social communication and interaction, plus at least two of four types of restricted repetitive behaviors (15). Mothers with complex medical, psychological, or mental disorders were excluded as these disorders may affect recall, self-administration, and outcome accuracy.

### 2.3 Sample size and sampling techniques

We determined the sample size using the formula n = Z^2^P(1-P)/d^2^, where n stands for the sample size, Z for the desired level of confidence, P for the expected prevalence, and d for the precision or effect size. Despite a 2008 surge in local research, much remains unexplored. Saudi Arabia’s ASD prevalence is unknown. One 2002 survey found 42,500 autistic cases. Recent comprehensive reviews found that ASD prevalence in Arabian Gulf nations, including Saudi Arabia (SA), ranged from 1.4 to 29 per 10,000 (16). With a 95% confidence level and 80% power, the calculated sample size was determined to be 88 mothers of autistic children. To collect the sample, the official groups used an online Delphi panel.

### 2.4 Data collection

#### 2.4.1 Data collection tool

A questionnaire was developed based on previous research (2, 4, 13–20). The questionnaire was initially drafted in English and then translated into Arabic. Two English-speaking translators performed the back translation, and they also consulted the original panel to address any issues or worries. To ensure content validity and reliability, the questionnaire underwent validation by three experts in public health, community medicine, and pediatrics. A survey was developed based on previous research (2, 4, 13–20). The questionnaire was initially drafted in English and then translated into Arabic. Two English-speaking translators performed the back translation, and they also consulted the original panel to address any issues or worries. To ensure content validity and reliability, the questionnaire underwent validation by three experts in public health, community medicine, and pediatrics. A pilot study was conducted on 10 participants who were not included in the main study to validate the questionnaire. We intentionally solicited feedback from participants, including inquiries about the clarity of the instructions, logistical, technical, and other concerns, as well as the difficulty of answering certain questions. After collecting and analyzing the pilot survey results, we addressed all the reported feedback, potentially correcting the questions or selecting the most appropriate types of queries. Cronbach’s alpha was calculated to estimate the questionnaire’s reliability, and a value of 0.78 was obtained.

The questionnaire is composed of five main sections, following the mothers’ written informed consent [found in **Supplementary Material 1**]. The first section includes the characteristics and demographic information of both the mother and father. 2) Prenatal risk factors for autism include health and pregnancy conditions, as well as adherence to required medications. 3) Antenatal risk factors include adherence to required follow-up, maternal factors such as environmental, psychosocial, and lifestyle-related dietary habits, and physical activity. We assessed psychosocial factors using the LIVES Daily Hassles score, a tool that evaluates the impact of frequent exposure to daily life stressors like family or marital conflict, income concerns, workload, time issues, concerns about future security, and environmental or housing conditions (20). 4) Natal-related risk factors. 5) The Autism Center uses the American Psychiatric Association’s DSM-5 classification, which provides information to the mothers regarding the diagnosis and severity of autism in their children (16).

#### 2.4.2 The Data collection process

Data collection was carried out using a pre-tested, pre-coded, and well-structured questionnaire. We designed the questionnaire in Arabic using Google Forms and distributed it to all registered mothers of autistic children in all autism centers, leveraging popular and officially recognized social media platforms such as Facebook, Twitter, mother-registered emails, and WhatsApp groups within an autism-specific center. We sent frequent reminder messages and follow-up communications to increase the response rate until we reached the desired sample size.

### 2.5 Statistical analysis

The Jamovi software program, version 2.5.6, was used for data coding and analysis. The Shapiro-Wilk test was employed to assess the normal distribution of the data. Qualitative data was presented as frequencies and percentages, while quantitative data was displayed as mean and standard deviation (SD) if it demonstrated normal distribution or as median and interquartile range (IQR) if it did not.

For examining the relationship between autism severity and continuous variables, we employed one-way ANOVA (Kruskal-Wallis). The chi-square test (χ2) and Fisher’s exact test were used to analyze the distinction between autism severity and other categorical variables, with Fisher’s exact test for sample sizes less than five and chi-square for larger sample sizes. Variables that demonstrated a significant association underwent multivariate logistic regression to assess their predictive capability for ASD severity. We considered the results statistically significant when the probability was less than 0.05.

### 2.6 Ethical considerations

The institutional review board (IRP) at Princess Nourah bint Abdulrahman University registered this survey under the number 24-0363 and gave its approval on February 19, 2024. Before participating in the Google forum, we requested that all invited participants complete and check an informed consent box. Participation in the survey was voluntary and related to the autism center. Checking the consent box and completing the voluntary survey were considered evidence of providing valid, informed consent. The IRB application for this study, which received approval, described this consent process.

## 3 Results

### 3.1 Regarding the frequency or severity of autism

Regarding the frequency or severity of autism, a survey of 168 mothers with children with ASD revealed that 15.6% of these children had mild autism, 31.9% had moderate autism, 43.8% had autism spectrum disorder, and 8.8% had severe autism.

### 3.2 Demographics and prenatal history, and their correlation with the severity grades of ASD

The median maternal age was 30 years, and the median paternal age was 35 years. Most participants resided in urban areas, 138 (86.3%), with slightly higher proportions in the mild and severe autism categories. In descending order regarding prenatal history or risk factors, 23 (14.4%) had ovarian cysts before pregnancy, 11 (6.9%) used assisted reproductive techniques (IVF-tubal pregnancy), 4 (2.5%) had type 1 diabetes, and 2 (1.3%) had type 2 diabetes before pregnancy. There was no statistically significant difference between the severity grades of ASD, demographics, and prenatal history (**Table 1**).

**Table 1:** Demographics and prenatal history and their relationship with the severity grades of ASD.

Variable	Total (n = 168) F (%)	Autism spectrum (n = 70,43.8%) F (%)	Mild autism (n = 25, 15.6%), F (%)	Moderate autism (n = 51, 31.9%) F (%)	Severe autism (n = 14,8.8%) F (%)	P value#
Mother’s age during pregnancy (years) Median (IQR)	30 (26-34)	30 (26-34)	30 (27.5-32)	29 (26.5-32.5)	30.5 (26.3-33.8),	0.999*
Father’s age during pregnancy (years) Median (IQR)	35 (30-42)	35 (31-38.8)	35 (29-40)	35 (30-46)	39 (30-45.8)	0.710*
Place of residence during pregnancy (n = 160)						
Rural	22 (13.8%)	13 (18.6%)	2 (8%)	6 (11.8%)	1 (7.1%)	0.563
Urban	138 (86.3%)	57 (81.4%)	23 (92%)	45 (88.2%)	13 (92.9%)	
Treatment for ovulation induction or assisted reproductive techniques (IVF – Tubal pregnancy) (n = 160)						
No	149 (93.1%)	66 (94.3%)	21 (84%)	49 (96.1%)	13 (92.9%)	0.248
Yes	11 (6.9%)	4 (5.7%)	4 (16%)	2 (3.9%)	1 (7.1%)	
Presence of ovarian cysts before pregnancy (n=160)						
No	114 (71.3%)	50 (71.4%)	16 (64%)	37 (72.5%)	11 (78.6%)	0.175
Yes	23 (14.4%)	11 (15.7%)	7 (28%)	4 (7.8%)	1 (7.1%)	
I don’t know§	23 (14.4%)	9 (12.9%)	2 (8%)	10 (19.6%)	2 (14.3%)	
Type 1 diabetes before pregnancy (n = 160)						
No	138 (86.3%)	66 (94.3%)	20 (80%)	40 (78.4%)	12 (85.7%)	0.121
Yes	4 (2.5%)	0 (0%)	1 (4%)	3 (5.9%)	0 (0%)	
I don’t know§	18 (11.3%)	4 (5.7%)	4 (16%)	8 (15.7%)	2 (14.3%)	
Type 2 diabetes before pregnancy (n = 160)						
No	140 (87.5%)	65 (92.9%)	21 (84%)	43 (84.3%)	11 (78.6%)	0.183
Yes	2 (1.3%)	1 (1.4%)	0 (0%)	0 (0%)	1 (7.1%)	
I don’t know§	18 (11.3%)	4 (5.7%)	4 (16%)	8 (15.7%)	2 (14.3%)	

F (Frequency), n = number of patients, IQR (Interquartile ranges: 25^th^ and 75^th^ percentiles), **#** (Fisher’s exact test), * (Kruksal-Wallis), **§**Excluded from the analysis.

### 3.3 The relationship between the severity grades of ASD and the antenatal maternal and paternal exposure history or maternal antenatal conditions (medical and environmental factors)

Most autistic children had a history of one or both maternal and/or paternal antenatal exposures, as follows, in descending order: 77.7% had soft drink consumption, 30.6% smoked, 20.7% had chronic physical organic diseases (e.g., high blood pressure, asthma), 16.9% had psychological or neurological disease, and 13.3% had a history of diet product consumption (aspartame).

Regarding maternal antenatal conditions, the most common events, in descending order, were: 33.7% history of recurrent infection; 25.7% anemia during pregnancy; and 26 (16.3%) threatened abortion or bleeding during pregnancy. During pregnancy, there is a risk of exposure to air pollution, which includes elevated carbon levels, 22 (13.8%) cases of gestational diabetes, 14 (10.7%) individuals taking medications for chronic conditions such as insulin, thyroxine, corticosteroids, and hydroxychloroquine, and 8 (5%) individuals receiving the COVID-19 vaccine and antibiotics such as amoxicillin, cotrimoxazole, and penicillin.

The study found no statistically significant difference between the severity grades of ASD, antenatal maternal and paternal exposure history, or maternal antenatal conditions (*p* > 0.05), except for gestational diabetes during pregnancy (*p* = 0.047). The results showed that 4 (28.6%) of 14 cases had severe autism, 8 (15.7%) of 50 cases had moderate autism, and 0 (0%) cases had mild autism. As illustrated in detail in **Table 2**.

**Table 2:** Antenatal maternal and paternal history and their relationship with the severity grades of ASD.

Variable	Total (n = 168) F (%)	Autism spectrum (n = 70,43.7%) F (%)	Mild ASD (n = 25, 15.6%) F (%)	Moderate ASD (n = 51, 31.9%) F (%)	Severe autism (n = 14,8.8%) F (%)	P value#
a) Maternal and/or paternal antenatal exposure						
Maternal smoking history (n = 157)						
Both	2 (1.3%)	2 (3%)	0 (0%)	0 (0%)	0 (0%)	0.828
Father only	46 (29.3%)	18 (26.9%)	9 (36%)	14 (27.5%)	5 (35.7%)	
None	109 (69.4%)	47 (70.1%)	16 (64%)	37 (72.5%)	9 (64.3%)	
Soft drink consumption (n = 157)						
Both	81 (51.6%)	42 (62.7%)	11 (44%)	20 (39.2%)	8 (57.1%)	0.225
Father only	27 (17.2%)	7 (10.4%)	6 (24%)	13 (25.5%)	1 (7.1%)	
Mother only	14 (8.9%)	6 (9%)	1 (4%)	5 (9.8%)	2 (14.3%)	
None	35 (22.3%)	12 (17.9%)	7 (28%)	13 (25.5%)	3 (21.4%)	
Diet soda consumption (Aspartame) (n = 157)						
Both	3 (1.9%)	1 (1.5%)	1 (4%)	1 (2%)	0 (0%)	0.776
Father only	9 (5.7%)	2 (3%)	1 (4%)	4 (7.8%)	2 (14.3%)	
Mother only	9 (5.7%)	5 (7.5%)	1 (4%)	3 (5.9%)	0 (0%)	
None	136 (86.6%)	59 (88.1%)	22 (88%)	43 (84.3%)	12 (85.7%)	
Had psychological, or neurological disease (n = 160)						
Both	2 (1.3%)	0 (0%)	0 (0%)	1 (2%)	1 (7.1%)	0.129
Father only	12 (7.5%)	5 (7.1%)	3 (12%)	1 (2%)	3 (21.4%)	
Mother only	13 (8.1%)	6 (8.6%)	2 (8%)	5 (9.8%)	0 (0%)	
None	133 (83.1%)	59 (84.3%)	20 (80%)	44 (86.3%)	10 (71.4%)	
Chronic physical organic diseases (e.g., high blood pressure, asthma) (n = 160)						
Both	2 (1.3%)	1 (1.4%)	1 (4%)	0 (0%)	0 (0%)	0.055
Father only	10 (6.3%)	1 (1.4%)	4 (16%)	4 (7.8%)	1 (7.1%)	
Mother only	21 (13.1%)	9 (12.9%)	5 (20%)	4 (7.8%)	3 (21.4%)	
None	127 (79.4%)	59 (84.3%)	15 (60%)	43 (84.3%)	10 (71.4%)	
b) Maternal antenatal conditions						
Preeclampsia during pregnancy (n = 160)						
No	155 (96.9%)	67 (95.7%)	24 (96%)	51 (100%)	13 (92.9%)	0.252
Yes	5 (3.1%)	3 (4.3%)	1 (4%)	0 (0%)	1 (7.1%)	
Gestational diabetes during pregnancy (n = 160)						
No	138 (86.3%)	60 (85.7%)	25 (100%)	43 (84.3%)	10 (71.4%)	0.047**
Yes	22 (13.8%)	10 (14.3%)	0 (0%)	8 (15.7%)	4 (28.6%)	
SARS-CoV-2 infection during pregnancy (n = 160)						
No	155 (96.9%)	68 (97.1%)	23 (92%)	51 (100%)	13 (92.9%)	0.125
Yes	5 (3.1%)	2 (2.9%)	2 (8%)	0 (0%)	1 (7.1%)	
COVID-19 vaccine received during pregnancy (n = 160)						
No	152 (95%)	66 (94.3%)	22 (88%)	51 (100%)	13 (92.9%)	0.07
Yes	8 (5%)	4 (5.7%)	3 (12%)	0 (0%)	1 (7.1%)	
Other vaccines received during pregnancy (n = 160)						
No	156 (97.5%)	67 (95.7%)	25 (100%)	51 (100%)	13 (92.9%)	0.199
Yes	4 (2.5%)	3 (4.3%)	0 (0%)	0 (0%)	1 (7.1%)	
Depression during pregnancy (n = 160)						
No	155 (96.9%)	67 (95.7%)	25 (100%)	50 (98%)	13 (92.9%)	0.513
Yes	5 (3.1%)	3 (4.3%)	0 (0%)	1 (2%)	1 (7.1%)	
Anemia during pregnancy (n = 160)						
No	119 (74.4%)	55 (78.6%)	16 (64%)	40 (78.4%)	8 (57.1%)	0.255
To some extent	11 (6.9%)	3 (4.3%)	2 (8%)	3 (5.9%)	3 (21.4%)	
Yes	30 (18.8%)	12 (17.1%)	7 (28%)	8 (15.7%)	3 (21.4%)	
Recurrent infections during pregnancy (n = 160)						
No	106 (66.3%)	42 (60%)	14 (56%)	39 (76.5%)	11 (78.6%)	0.386
To some extent	25 (15.6%)	14 (20%)	4 (16%)	6 (11.8%)	1 (7.1%)	
Yes	29 (18.1%)	14 (20%)	7 (28%)	6 (11.8%)	2 (14.3%)	
Threatened abortion or bleeding during pregnancy (n = 160)						
No	134 (83.8%)	61 (87.1%)	23 (92%)	38 (74.5%)	12 (85.7%)	0.194
Yes	26 (16.3%)	9 (12.9%)	2 (8%)	13 (25.5%)	2 (14.3%)	
Exposure to air pollution during pregnancy (such as increased carbon levels) (n = 160)						
No	150 (93.8%)	66 (94.3%)	24 (96%)	50 (98%)	14 (100%)	0.952
To some extent	4 (2.5%)	1 (1.4%)	1 (4%)	1 (2%)	0 (0%)	
Yes	6 (3.8%)	3 (4.3%)	0 (0%)	0 (0%)	0 (0%)	
Exposure to high doses of lead or arsenic during pregnancy (n = 160)						
No	154 (96.3%)	66 (94.3%)	24 (96%)	50 (98%)	14 (100%)	0.661
To some extent	3 (1.9%)	1 (1.4%)	1 (4%)	1 (2%)	0 (0%)	
Yes	3 (1.9%)	3 (4.3%)	0 (0%)	0 (0%)	0 (0%)	
Medication use during pregnancy (n = 131)						
Antibiotics (e.g. Amoxicillin, Co-trimoxazole, Penicillin)	8 (6.1%)	2 (3.2%)	3 (15.8%)	2 (5.3%)	1 (8.3%)	0.942
Antidepressants	3 (2.3%)	2 (3.2%)	0 (0%)	1 (2.6%)	0 (0%)	
Medications Specifically for Pregnancy Management (e.g. Progesterone, Heparin, Aspirin, Folgard, Diclofenac)	7 (5.3%)	3 (4.8%)	0 (0%)	3 (7.9%)	1 (8.3%)	
Medications for Chronic Conditions (e.g. Insulin, Thyroxine, Corticosteroids, Hydroxychloroquine)	14 (10.7%)	5 (8.1%)	3 (15.8%)	5 (13.2%)	1 (8.3%)	
Medications for Nausea and Heartburn (e.g. Antacids, Domperidone, Omeprazole)	4 (3.1%)	2 (3.2%)	1 (5.3%)	1 (2.6%)	0 (0%)	
Prenatal Vitamins and Supplements (e.g. Folic acid, Calcium, Iron, Vitamin B12, Vitamin D)	9 (6.9%)	5 (8.1%)	1 (5.3%)	3 (7.9%)	0 (0%)	
No medications	82 (62.6%)	41 (66.1%)	11 (57.9%)	21 (55.3%)	9 (75%)	
I don’t remember§	4 (3.1%)	2 (3.2%)	0 (0%)	2 (5.3%)	0 (0%)	

**Statistically significant difference, **§**Excluded from the analysis.

### 3.4 Maternal lifestyle factors (including dietary and physical activity during pregnancy) and their relationship with the severity grades of ASD

Although approximately 45% of mothers didn’t remember their average daily intake, the majority reported less than the daily recommended diet of milk and dairy products. 67, 41.9%/94: vegetables 66, 41.3%/91: fruit 65, 65, 40.6%/93: water consumption 115, 71.9%): grains and bread 40, 25%/64; meat and legumes 43, 26.9%/85. The level of physical activity was 102 (63.7%). Routine household activities. The study found no significant association between the severity of ASD and the nutrition and lifestyle factors during pregnancy. As illustrated in detail in **Table 3**.

**Table 3:** Maternal lifestyle factors (including dietary and physical activity during pregnancy) and their relationship with the severity grades of ASD.

Variable	Total (n = 168) F (%)	Autism spectrum (n=70,43.8%) F (%)	Mild ASD (n = 25, 15.6%) F (%)	Moderate ASD (n = 51, 31.9%) F (%)	Severe ASD (n = 14,8.8%) F (%)	*P* value
Servings of grains and bread consumed daily (One serving equals one slice of bread - 25 grams - or half a cup of cooked grains or breakfast cereals, or 4-6 medium-sized biscuits) (n = 160)						
Less than 6 servings	40 (25%)	17 (24.3%)	7 (28%)	11 (21.6%)	5 (35.7%)	0.678
6 to 11 servings	22 (13.8%)	10 (14.3%)	4 (16%)	7 (13.7%)	1 (7.1%)	
More than 11 servings	2 (1.3%)	1 (1.4%)	0 (0%)	0 (0%)	1 (7.1%)	
I don’t know/remember §	96 (60%)	42 (60%)	14 (56%)	33 (64.7%)	7 (50%)	
Servings of meat and legumes consumed daily (One serving equals 60-90 grams of red meat, chicken, fish, or half a cup of cooked legumes) (n = 160)						
Less than 2 servings	43 (26.9%)	15 (21.4%)	7 (28%)	16 (31.4%)	5 (35.7%)	0.787
2 to 3 servings	32 (20%)	16 (22.9%)	4 (16%)	9 (17.6%)	3 (21.4%)	
More than 3 servings	10 (6.3%)	3 (4.3%)	2 (8%)	3 (5.9%)	2 (14.3%)	
Never consume (vegetarian)	2 (1.3%)	1 (1.4%)	1 (4%)	0 (0%)	0 (0%)	
I don’t know/remember §	73 (45.6%)	35 (50%)	11 (44%)	23 (45.1%)	4 (28.6%)	
Servings of milk and dairy products consumed daily (One serving equals one cup of milk or yogurt - 240 ml - or 30 grams of cheese) (n = 160)						
Less than 3 servings	67 (41.9%)	28 (40%)	10 (40%)	21 (41.2%)	8 (57.1%)	0.111
3 to 5 servings	23 (14.4%)	10 (14.3%)	4 (16%)	7 (13.7%)	2 (14.3%)	
More than 5 servings	4 (2.5%)	2 (2.9%)	0 (0%)	2 (3.9%)	0 (0%)	
I don’t know/remember §	66 (41.3%)	30 (42.9%)	11 (44%)	21 (41.2%)	4 (28.6%)	
Servings of vegetables consumed daily (One serving equals one cup of raw vegetables or half a cup of vegetable juice or half a cup of cooked vegetables) (n = 160)						
Less than 3 servings	66 (41.3%)	26 (37.1)	10 (40%)	23 (45.1%)	7 (50%)	0.869
3 to 5 servings	19 (11.9%)	6 (8.6%)	3 (12%)	6 (11.8%)	4 (28.6%)	
More than 5 servings	6 (3.8%)	2 (2.9%)	1 (4%)	3 (5.9%)	0 (0%)	
I don’t know/remember §	69 (43.1%)	36 (51.4%)	11 (44%)	19 (37.3%)	3 (21.4%)	
Servings of fruit consumed daily (One serving equals one medium-sized apple, orange, or banana, or half a cup - 120 ml - of fruit juice or dried fruit) (n = 160)						
Less than 2 servings	65 (40.6%)	27 (38.6%)	10 (40%)	22 (43.1%)	6 (42.9%)	0.994
2 to 3 servings	23 (14.4%)	9 (12.9%)	3 (12%)	8 (15.7%)	3 (21.4%)	
More than 3 servings	5 (3.1%)	2 (2.9%)	1 (4%)	2 (3.9%)	0 (0%)	
I don’t know/remember §	67 (41.9%)	32 (45.7%)	11 (44%)	19 (37.3%)	5 (35.7%)	
Cups of water consumed daily (One cup is 240 milliliters) (n = 160)						
Less than 7 cups	115 (71.9%)	53 (75.7%)	17 (68%)	35 (68.6%)	10 (71.4%)	0.817
7 to 9 cups	40 (25%)	15 (21.4%)	8 (32%)	13 (25.5%)	4 (28.6%)	
More than 9 cups	5 (3.1%)	2 (2.9%)	0 (0%)	3 (5.9%)	0 (0%)	
**Total consumption score Median [IQR] (n = 160)**			5 [1-8]	5 [2-7]	6 [3.5-9]	0.678
The level of physical activity during pregnancy (n = 160)						
Basic daily needs	37 (23.1%)	16 (22.9%)	7 (28%)	11 (21.6%)	3 (21.4%)	0.565
Routine household activities	102 (63.7%)	47 (67.1%)	12 (48%)	32 (62.7%)	11 (78.6%)	
I engage in walking (half an hour, three to four times a week) or moderate physical activity	15 (9.4%)	6 (8.6%)	4 (16%)	5 (9.8%)	0 (0%)	
I engage in more physical activity (such as walking an hour and a half per week) or vigorous physical activity	6 (3.8%)	1 (1.4%)	2 (8%)	3 (5.9%)	0 (0%)	

I don’t know: 0; Less than the recommended servings: 1; Within the recommended range of servings: 2; More than the recommended servings: 3; Median [Q1, Q3]; One-Way ANOVA (Kruskal-Wallis). § Excluded from the analysis//.

(Grains and bread, Meat and legumes, Milk and dairy products, Vegetables, Fruits, Water).

### 3.5 LIVES Daily Hassles Factors and Scale (psycho-social factors) during pregnancy and their relationship with the severity grades of ASD

In descending order, the majority reported that daily hassles, including household chores, spouse conflict, time pressure, financial concerns, internal fears, and work-related factors, had a negative impact on their health. The study found that there was no significant correlation between the severity of autism spectrum disorder (ASD) and the LIVES Daily Hassles Factors and Scale during pregnancy. However, the impact of household chores on health during pregnancy was significantly (P = 0.02) higher among individuals with severe autism (around 14.3%), compared to those with mild autism (12%) or moderate autism (5.9%). As illustrated in detail in **Table 4**.

**Table 4:** LIVES daily hassles factors and scale during pregnancy and their relationship with the severity grades of ASD.

Variable	Total F (%)	Autism spectrum (n=70,43.8%) F (%)	Mild ASD (n = 25, 15.6%) F (%)	Moderate ASD (n = 51, 31.9%) F (%)	Severe ASD (n = 14,8.8%) F (%)	*P* value
Impact of internal fears on health during pregnancy (fears of being alone, fear of confrontation) (n = 160)						
Very negative impact	24 (15%)	11(15.7%)	3 (12%)	9 (17.6%)	1 (7.1%)	0.627
Negative impact	23 (14.4%)	13(18.6%)	3 (12%)	7 (13.7%)	0 (0%)	
There is, but it does not have an effect	32 (20%)	16(22.9%)	5 (20%)	7 (13.7%)	4 (28.6%)	
None at all	81 (50.6%)	30(42.9%)	14 (56%)	28 (54.9%)	9 (64.3%)	
Impact of household chores on health during pregnancy (n = 160)						
Very negative impact	15 (9.4%)	7 (10%)	3 (12%)	3 (5.9%)	2 (14.3%)	0.02**
Negative impact	30 (18.8%)	14 (20%)	2 (8%)	14 (27.5%)	0 (0%)	
There is, but it does not have an effect	79 (49.4%)	36(51.4%)	18 (72%)	20 (39.2%)	5 (35.7%)	
None at all	36 (22.5%)	13(18.6%)	2 (8%)	14 (27.5%)	7 (50%)	
Impact of Spouse conflict on health during pregnancy (n = 160)						
Very negative impact	29 (18.1%)	14 (20%)	4 (16%)	9 (17.6%)	2 (14.3%)	0.233
Negative impact	24 (15%)	10(14.3%)	2 (8%)	11 (21.6%)	1 (7.1%)	
There is, but it does not have an effect	61 (38.1%)	29(41.4%)	14 (56%)	15 (29.4%)	3 (21.4%)	
None at all	46 (28.7%)	17(24.3%)	5 (20%)	16 (31.4%)	8 (57.1%)	
Impact of time pressure on health during pregnancy (n = 160)						
Very negative impact	21 (13.1%)	10(14.3%)	3 (12%)	6 (11.8%)	2 (14.3%)	0.537
Negative impact	34 (21.3%)	15(21.4%)	5 (20%)	13 (25.5%)	1 (7.1%)	
There is, but it does not have an effect	51 (31.9%)	27(38.6%)	5 (20%)	15 (29.4%)	4 (28.6%)	
None at all	54 (33.8%)	18(25.7%)	12 (48%)	17 (33.3%)	7 (50%)	
Impact of environmental or housing conditions on health during pregnancy (neighbors, crime, deterioration) (n = 160)						
Very negative impact	5 (3.1%)	2 (2.9%)	3 (12%)	0 (0%)	0 (0%)	0.174
Negative impact	7 (4.4%)	3 (4.3%)	2 (8%)	1 (2%)	1 (7.1%)	
There is, but it does not have an effect	21 (13.1%)	9 (12.9%)	3 (12%)	9 (17.6%)	0 (0%)	
None at all	127 (79.4%)	56 (80%)	17 (68%)	41 (80.4%)	13 (92.9%)	
Impact of financial concerns on health during pregnancy (n = 160)						
Very negative impact	23 (14.4%)	5 (7.1%)	6 (24%)	11 (21.6%)	1 (7.1%)	0.145
Negative impact	21 (13.1%)	6 (8.6%)	4 (16%)	9 (17.6%)	2 (14.3%)	
There is, but it does not have an effect	38 (23.8%)	21 (30%)	4 (16%)	11 (21.6%)	2 (14.3%)	
None at all	78 (48.8%)	38(54.3%)	11 (44%)	20 (39.2%)	9 (64.3%)	
Impact of work-related factors on health during pregnancy (issues with colleagues or job dissatisfaction) (n = 160)						
Very negative impact	5 (3.0%)	1 (1.4%)	2 (8%)	2 (3.9%)	0 (0%)	0.383
Negative impact	14 (8.3%)	5 (7.1%)	4 (16%)	5 (9.8%)	0 (0%)	
There is, but it does not have an effect	20 (11.9%)	11(15.7%)	2 (8%)	7 (13.7%)	0 (0%)	
None at all	121 (72.0%)	53(75.7%)	17 (68%)	37 (72.5%)	14 (100%)	
Impact of concerns about future security on health during pregnancy (job stability, inability to work) (n = 160)						
Very negative impact	8 (5%)	3 (4.3%)	2 (8%)	3 (5.9%)	0 (0%)	0.939
Negative impact	13 (8.1%)	7 (10%)	1 (4%)	5 (9.8%)	0 (0%)	
There is, but it does not have an effect	18 (11.3%)	8 (11.4%)	2 (8%)	7 (13.7%)	1 (7.1%)	
None at all	121 (75.6%)	52(74.3%)	20 (80%)	36 (70.6%)	13 (92.9%)	
**LIVES Daily Hassles Scale ¥ Median [IQR] (n = 160)**		6 [3-9]	5 [2-11]	7 [2.5-11]	3 [0-6.5]	0.170

¥None at all: 0; There is, but it does not have an effect: 1; Negative impact: 2; Very negative impact: 3; Median [Q1, Q3]; One-Way ANOVA (Kruskal-Wallis). ** Statistically significant difference.

### 3.6 The natal history, ASD diagnosis history, and correlation with ASD severity grades

In terms of natal history and risk factors, there were 104 (65%) autistic children born in the government hospital. Out of these, 97 (60.6%) were born vaginally, 21.2% had an abnormal birth weight, 50 (31.3%) required admission to the neonatal intensive care unit, 23 (14.4%) had a history of oxygen desaturation, 11.9% had an abnormal birth length, and 16 (10%) had a history of any or recurrent infections. Only 88 (55%) cases with a history of autism received a diagnosis based on symptoms, with 82 (51.2%) families serving as the primary caregivers.

The study found no significant correlation between the severity of ASD and the natal history and ASD diagnosis. The study did, however, find a strong link (P = 0.021) between a child’s length at birth—which was higher at 3 (21.4%) in cases of severe autism compared to other grades less than 10%—and their oxygen desaturation—which was higher at 6 (42.9%) in cases of severe autism compared to other grades less than 14.3% (p = 0.017). In addition, the current median age of the child (years) is significantly (*p* = 0.035) higher in severe autism cases at 14 (12.25–18) compared to the median mild autism 7 (5.5–13), compared to the median moderate as well as autism spectrum. As illustrated in detail in **Table 5**.

**Table 5:** The natal history and ASD diagnosis history and their relationship with the severity grades of ASD.

Variable	Total (n = 168) F (%)	Autism spectrum (n=70,43.8%) F (%)	Mild ASD (n = 25, 15.6%) F (%)	Moderate ASD (n = 51, 31.9%) F (%)	Severe ASD (n = 14,8.8%) F (%)	*P* value
**Gestational age (weeks) at delivery** Median (IQR) **(n = 142**)	36 (36-38)	36 (36-37)	36 (36-38)	37 (36-38)	36 (32.8-36.5)	0.214
Place of birth (n = 160)						
At clinic	1 (0.6%)	0 (0%)	0 (0%)	1 (2%)	0 (0%)	0.084
At home	1 (0.6%)	0 (0%)	1 (4%)	0 (0%)	0 (0%)	
Government hospital	104 (65%)	50 (71.4%)	11 (44%)	32 (62.7%)	11 (78.6%)	
Private hospital	54 (33.8%)	20 (28.6%)	13 (52%)	18 (35.3%)	3 (21.4%)	
Type of delivery (n = 160)						
Cesarean section	47 (29.4%)	20 (28.6%)	11 (44%)	13 (25.5%)	3 (21.4%)	0.533
Cesarean section with complications or difficulties	16 (10%)	4 (5.7%)	4 (16%)	6 (11.8%)	2 (14.3%)	
Vaginal delivery	61 (38.1%)	30 (42.9%)	6 (24%)	19 (37.3%)	6 (42.9%)	
Vaginal delivery with complications or difficulties	36 (22.5%)	16 (22.9%)	4 (16%)	13 (25.5%)	3 (21.4%)	
Anesthesia during childbirth (n = 160)						
Local anesthesia	12 (7.5%)	4 (5.7%)	3 (12%)	2 (3.9%)	3 (21.4%)	0.267
Partial anesthesia	35 (21.9%)	14 (20%)	9 (36%)	11 (21.6%)	1 (7.1%)	
Full anesthesia	33 (20.6%)	15 (21.4%)	5 (20%)	10 (19.6%)	3 (21.4%)	
No	80 (50%)	37 (52.9%)	8 (32%)	28 (54.9%)	7 (50%)	
Child’s weight at birth (n = 160)						
Above normal	13 (8.1%)	5 (7.1%)	2 (8%)	5 (9.8%)	1 (7.1%)	0.376
Normal	119 (74.4%)	52 (74.3%)	19 (76%)	40 (78.4%)	8 (57.1%)	
Below normal	21 (13.1%)	11 (15.7%)	3 (12%)	3 (5.9%)	4 (28.6%)	
I don’t know/remember §	7 (4.4%)	2 (2.9%)	1 (4%)	3 (5.9%)	1 (7.1%)	
Child’s length at birth (n = 160)						
Above normal	6 (3.8%)	3 (4.3%)	2 (8%)	0 (0%)	1 (7.1%)	0.021**
Normal	126 (78.8%)	52 (74.3%)	18 (72%)	48 (94.1%)	8 (57.1%)	
Below normal	13 (8.1%)	7 (10%)	2 (8%)	1 (2%)	3 (21.4%)	
I don’t know/remember §	15 (9.4%)	8 (11.4%)	3 (12%)	2 (3.9%)	2 (14.3%)	
Neonatal Intensive care unit admission (n = 160)						
No	110 (68.8%)	49 (70%)	15 (60%)	37 (72.5%)	9 (64.3%)	0.671
Yes	50 (31.3%)	21 (30%)	10 (40%)	14 (27.5%)	5 (35.7%)	
Child had a history of any or recurrent infections (n = 160)						
No	144 (90%)	66 (94.3%)	21 (84%)	45 (88.2%)	12 (85.7%)	0.319
Yes	16 (10%)	4 (5.7%)	4 (16%)	6 (11.8%)	2 (14.3%)	
Child having any seizures (n = 160)						
No	154 (96.3%)	64 (91.4%)	25 (100%)	51 (100%)	14 (100%)	0.081
Yes	6 (3.8%)	6 (8.6%)	0	0	0	
Child having a history oxygen desaturation (n = 160)						
No	137 (85.6%)	60 (85.7%)	24 (96%)	45 (88.2%)	8 (57.1%)	0.017**
Yes	23 (14.4%)	10 (14.3%)	1 (4%)	6 (11.8%)	6 (42.9%)	
First person to hold the baby (suspect abnormality) (n = 160)						
Doctor	78 (48.8%)	33 (47.1%)	9 (36%)	28 (54.9%)	8 (57.1%)	0.418
Family	82 (51.2%)	37 (52.9%)	16 (64%)	23 (45.1%)	6 (42.9%)	
Method of diagnosis (n = 160)						
According to diagnostic criteria (by a multidisciplinary medical team)	43 (26.9%)	15 (21.4%)	6 (24%)	17 (33.3%)	5 (35.7%)	0.216
According to diagnostic criteria (with radiological and laboratory tests)	29 (18.1%)	16 (22.9%)	1 (4%)	9 (17.6%)	3 (21.4%)	
Based on symptoms only	88 (55%)	39 (55.7%)	18 (72%)	25 (49%)	6 (42.9%)	
**The current age (years) of the child** **Median (IQR) (n = 168)**	9 (6-14)	9 (6-14)	7 (5.5-13)	9 (6-14)	14 (12.25-18)	0.035**

**Statistically significant difference, **§**Excluded from the analysis.

### 3.7 The predictors of severe ASD by ordinal logistic regression

#### 3.7.1 In terms of antenatal history

In terms of antenatal history, gestational diabetes during pregnancy is a strong predictor of severe autism. The aOR for the severity of ASD among women with gestational diabetes during pregnancy relative to those without gestational diabetes during pregnancy is 4.553 (95% CI: [1.518, 14.25], *P* = 0.008) (**Table 6**). This suggests that women with gestational diabetes during pregnancy are approximately 4.5 times more likely to have children with severe ASD compared to those without gestational diabetes during pregnancy.

**Table 6:** The predictors of severe ASD by ordinal logistic regression.

Variable	aOR	95% CI		*P* value
		Lower	Upper	
Chronic physical organic diseases (e.g., high blood pressure, asthma)					
Father only vs. Both	0.545	0.021	19.22	0.707
Mother only vs. Both	2.354	0.109	74.16	0.578
None vs. Both	5.037	0.252	147.27	0.277
Gestational diabetes during pregnancy					
Yes vs. No	4.553	1.518	14.25	0.008**
Impact of household chores on health during pregnancy					
None at all vs. Negative impact	1.435	0.554	3.73	0.456
There is, but it does not have an effect vs. Negative impact	0.792	0.343	1.81	0.581
Very negative impact vs. Negative impact	1.188	0.352	4.01	0.78
Child’s length at birth					
Below normal vs. Above normal	1.808	0.209	15.71	0.588
Normal vs. Above normal	1.158	0.188	6.92	0.872
Child having oxygen desaturation					
Yes vs. No	4.142	1.437	12.45	0.01**
The current age of the child (years)	1.071	1.002	1.15	0.045**

**Statistically significant difference.

#### 3.7.2 Regarding natal history a child’s oxygen desaturation

Regarding natal history A child’s oxygen desaturation is a significant predictor of severe autism. The aOR for the severity of ASD among children with oxygen desaturation relative to those without oxygen desaturation is 4.142 (95% CI: [1.437, 12.45], P = 0.01). This suggests that children with oxygen desaturation are approximately 4.1 times more likely to develop severe ASD than those without.

#### 3.7.3 In terms of autism history

In terms of autism history, a child’s current age in years is a significant predictor of severe autism. The aOR for the severity of ASD associated with each additional year of the child’s age is 1.071 (95% CI: [1.002, 1.15], *P* = 0.045). This indicates that for every one-year increase in the child’s age, the odds of being in a higher category of ASD severity relative to a lower category increase by approximately 7.1%.

## 4 Discussion

In SA, there is a significant gap between the demands of those diagnosed with ASD and the currently offered services (17). Early intervention focuses on language, play skills, social involvement, activities of daily living (ADLs), and disruptive behaviors. Genetic and epigenetic factors may contribute to ASD’s multifactorial origin (21). As a result, this local study from SA is critical to highlighting prenatal, antenatal, and natal-related factors, or associated ASD.

### 4.1 The frequency or severity of autism

In this study, the majority of the recruited 168 mothers with Autistic children reported having autism spectrum disorders (43.8%). Moderate autism accounts for 31.9%, mild autism for 15.6%, and severe autism (8.8%). According to the global classification of ASD severity, most mothers reported having ASD, a broader category that encompasses all levels of severity, including moderate, mild, and severe autism. Remember, diagnostic practices are crucial.

Rates of occurrence may be different depending on diagnostic criteria, cultural contexts, and research methods; without detailed sub-categorization, this isn’t standard practice (22). For example, a narrative review of UK clinical practice guidelines for ASD diagnosis shows that diagnostic rates vary depending on social and contextual factors (23). Furthermore, the level of maternal awareness and understanding were relatively low, with only 56.2% being able to identify and comprehend their child’s subcategorical classification.

### 4.2 Demographics and prenatal history and their relationship with the severity grades of ASD

#### 4.2.1 Parental age and ASD

Although there was a non-significant association between parental age and ASD severity, those with severe autism had a relatively older median (IQR) parent age of 39 years (30–45.8), while other groups were 35 years. This may be due to the relatively small number of severe autism cases (8.8%). Previous research links older parental age to higher ASD risk in children, potentially due to genetic mutations in older gametes and exposure to various environmental factors over a longer period, some of which could impact the health and development of their offspring (5–24).

#### 4.2.2 Polycystic ovarian syndrome

We found that 23 mothers (13.7%) of children with ASD had history of PCOS, with an average of 5% to 20% of PCOS among women of reproductive age and no significant correlation with the severity grades. In agreement with a large-scale study from Sweden, PCOS has identified a potential increased risk of birthing children with ASD. Although PCOs underlying pathogenic mechanisms have not been completely understood, prenatal hyperandrogenism (male sex hormones; chronic anovulation, insulin resistance, metabolic syndrome, chronic low-grade inflammation)—all these factors may affect the intrauterine environment and may play a role in neurodevelopmental disorders, including ASD (6–25).

### 4.3 Antenatal maternal and paternal exposure history or maternal antenatal conditions and their relationship with the severity grades of ASD

#### 4.3.1 Soft drink consumption

Out of 168 mothers (8.3%), 81 (48.2%) reported that both fathers and mothers drank soft drinks, and 19% had a history of diet product consumption (aspartame). The FDA approved aspartame as a tabletop sweetener in 1981. By 1983, the FDA had reported many adverse events, e.g., depression, anxiety, headache complaints, and other neurological problems, including irritability, mood disorders, cognitive problems, and seizures (26, 27).

Moreover Aspartame-fed animals displayed cognitive problems, anxiety-related behaviors, and other noticeable neurophysiologic effects. This has led to the discovery of long-term health problems in humans and their offspring, as the availability of glutamine sulfhydryl (GSH) has greatly decreased. This is significant because GSH protects the developing brain by scavenging toxins, preventing oxidative stress, and supporting methylation. There are more free radicals, oxidative stress, lipid peroxidation, inflammation, mitochondrial dysfunction, excitotoxicity, neuronal apoptosis, serotonin, noradrenaline, and dopamine in the brain when aspartame and its byproducts are present (27–29).

#### 4.3.2 Maternal smoking history

Maternal smoking history: 35.1% out of 168 mothers reported that fathers were smoking. The adjusted odds ratio (aOR) indicates the strength of this association, as well as a higher likelihood of having offspring with ASD (11). In agreement with other systematic reviews, tobacco smoke is the poisonous substance that most likely harms brain development. Nicotine affects nicotinic acetylcholine receptors in the offspring later in life, which mediate neural structural changes that can lead to adverse birth outcomes such as fetal growth restriction and low birth weight. Moreover, it significantly alters sperm DNA methylation and gene expression (11).

#### 4.3.3 Medication use during pregnancy

Medication use during pregnancy We did not identify a notable association between medication use during pregnancy and ASD; however, other studies have suggested that the use of certain substances might be a risk factor for ASD. For instance, a study suggested that the use of antidepressants, specifically selective serotonin reuptake inhibitors, during the second and/or third trimesters increases the risk of ASD (8).

Among ASD mothers, only 9 (5.4%) consume prenatal vitamins and supplements (e.g., folic acid, calcium, iron, vitamin B12, and vitamin D). This indicates that consuming prenatal vitamins and supplements reduces the risk of ASD, and certain nutrients may have neuroprotective effects throughout development. This aligns with a systematic review that found that maternal prenatal vitamins, folic acid, and vitamin D reduce the risk of autism in offspring. Higher maternal intakes of folic acid, omega-6 fatty acids, and vitamin D during pregnancy reduced child autism risk, while women deficient in omega-3 and polyunsaturated fatty acids raised it (30, 31).

#### 4.3.4 Infections and anemia during pregnancy

During pregnancy, 29 out of 168 mothers (17.3%) reported having recurrent infections. The nested case-control study found no overall significant association between any maternal infection during pregnancy and infections diagnosed during hospital admission, particularly bacterial infections (32). The lower level of immunity, along with the associated anemia, may pose a potential risk for ASD in 30 out of 168 mothers (17.9%). In alignment with other research, mothers diagnosed with anemia within the first 30 weeks of pregnancy had a higher prevalence of ASD, ADHD, and intellectual disabilities (33, 34).

#### 4.3.5 The study examines the relationship between maternal lifestyle factors (such as dietary and physical activity during pregnancy) and severity grades

##### 4.3.5.1 Nutrition during pregnancy

Although approximately 45% of mothers didn’t remember their average daily intake, the majority reported less than the daily recommended diet of milk and dairy products. 67, 78.7%/94: vegetables 66, 72.5%/91: fruit. 65, 69.9%/93: water, followed by grains and bread at 40, 60%/64, and meat and legumes at 45, 51.7%/87. This recall may indicate that the mothers did not follow an appropriate diet, which could potentially influence the risk of ASD. This pattern of dietary consumption is consistent with another two studies in SA (35, 36). This result aligns with the findings of another two United States cohorts, which suggested a positive association between the Western diet and autism-related traits (37).

Here are some key points highlighting the importance of nutrition during pregnancy: 1. Fetal Development: Adequate intake of nutrients such as folic acid, iron, calcium, and omega-3 fatty acids supports the development of the baby’s brain, bones, and overall body structure. 2. Birth Defect Prevention: Proper nutrition can help prevent birth defects. For instance, folic acid is known to reduce the risk of neural tube defects. 3. Healthy Birth Weight: A balanced diet helps to achieve a healthy birth weight, reducing the risk of complications during delivery and ensuring the baby has a favorable start in life. 4. Immune System Support: Nutrients like vitamins A, C, and E, along with zinc, support the development of the baby’s immune system, helping to protect against infections. 5. Long-term Health: Maternal nutrition can have long-term effects on the child’s health, influencing their risk of developing chronic conditions such as obesity, diabetes, and cardiovascular diseases later in life. 6. Cognitive Development: Essential fatty acids, particularly DHA, are critical for the development of the baby’s brain and eyes, potentially impacting cognitive function and vision (12).

##### 4.3.5.2 The study focuses on the Daily Hassles Factors, their scale during pregnancy, and their relationship with the severity grades of ASD

In descending order, the majority reported that there was a negative impact on the health of the daily hassles: 78.5% of household chores, 72.6% of spouse conflict, 67.8% of time pressure, 53.6% of financial concerns, 51.8% of internal fears, and 27.9% of concerns about the future, as well as work-related factors. There was no significant correlation between the severity of ASD and the LIVES Daily Hassles Factors and Scale during pregnancy, according to the study. However, the impact of household chores on health during pregnancy was significantly (*P* = 0.02) higher among individuals with severe autism (around 14.3%), compared to those with mild autism (12%) or moderate autism (5.9%). Household chores and pregnancy stress: In our study, we identified a notable association between the stress from household chores during pregnancy and ASD development. This underscores the potential impact of prenatal stress on fetal development, possibly through hormonal changes or other stress responses. Our findings call for more support for expectant mothers to reduce stress and physical strain, which may lower ASD risk (38, 39).

### 4.4 The history of ASD diagnosis and its relationship to ASD severity grades

Two major domains of ASD diagnosis are social and communication interaction and restricted or repetitive conduct. In this study, only 88 (55%) cases received a diagnosis based on their symptoms, while 82 (51.3%) families handled the diagnosis. SA-related methodological issues, poor diagnostic competence, and lower parent awareness of ASD can all contribute to this gap, limiting the likelihood of recognizing symptoms and seeking therapy. Parents of autistic children face a lack of government and institutional support, as well as a lack of resources for autistic children, which may affect other siblings’ quality of life, their social and economic lives, family ties. In Saudi Arabia, 88.5% of parents of children with autism experience discomfort; out of 61 families with ASD, 37% felt ashamed for having autistic children, and 64% were concerned about others’ treatment. According to SA Khan et al.’s 2020 cross-sectional study, only 31% of children on the autistic spectrum could attend neighboring autism clinics, and 72% had no access to private ASD schools (40–42).

Radiological and laboratory tests diagnosed only 29 (18.1%) ASD cases, primarily diagnosing 16 (55%) cases of autism spectrum disorder. While SA recommends genetic testing for all ASD patients, chromosomal microarray analysis (CMA) is more effective when the cause of developmental delay is unknown, as 20-30% of individuals on the autism spectrum exhibit significant genetic variation and new mutations. Moreover, consanguinity in Saudi culture—57.7% of marriages—was considered in these recommendations (43, 44).

### 4.5 Factors associated with the severity of autism

#### 4.5.1 Gestational diabetes mellitus

In this study, 22 out of 168 mothers (13.1%) reported having GDM, and there was a statistically significant association (*p*-value of 0.047) between GDM and the severity of ASD. Numerous mechanisms explain this association and GDM’s role. 1) GDM influences placental function, which in turn alters the delivery of nutrients and oxygen to the fetus, leading to hypoxia. Hypoxia (the developing brain doesn’t receive enough oxygen) can impair neurogenesis and lead to neuronal cell death, thereby contributing to neurodevelopmental impairments and potentially increasing the severity of ASD. Hypoxia can lead to high blood sugar levels, which can cause oxidative stress and inflammation in the developing fetal brain. 2) High blood sugar increases the production of reactive oxygen species, leading to oxidative stress, which can harm cellular structures such as lipids, proteins, and DNA, potentially disrupting normal brain development. 3) High blood sugar-induced inflammation can trigger the release of pro-inflammatory cytokines, potentially disrupting neuronal development and synaptic plasticity. These inflammatory processes can worsen the severity of ASD by further impairing neural connectivity and function (10, 45, 46). Moreover, this underscores the importance of closely monitoring and managing gestational diabetes during pregnancy.

#### 4.5.2 Oxygen desaturation and ASD

Children exposed to oxygen desaturation at birth are more likely to develop severe ASD, according to a *p*-value of 0.017. This aligns with previous studies (45–49). This finding highlights the importance of early interventions for children with perinatal complications to potentially reduce ASD severity (47, 50). However, researchers are still exploring the exact pathological mechanisms that link oxygen desaturation to ASD. Hypoxia can cause inflammation, oxidative stress, and brain development disruptions, which may contribute to the development of ASD (4, 51).

#### 4.5.3 Child’s age and ASD severity level

A 7.1% increase in the likelihood of classifying a child’s ASD as more severe with each year. This suggests that the challenges associated with ASD may become more pronounced as children age. However, it’s important to note that ASD symptoms can change over time, with some children showing a decrease in severity. This variability indicates that ASD is a dynamic condition, and interventions should be adaptable to each child’s changing needs (48).

### 4.6 Strengths and limitations

This study has many strengths, including the relatively large sample size, the use of a validated tool, the collection of paternal and maternal factors, and the comprehensiveness of prenatal, antenatal, and natal medical, environmental, dietary, physical, and psychosocial factors. Furthermore, we investigated the effect of frequent exposure to seven daily life stressors on health, which include family or marital conflict, income concerns, workload, time issues, concerns about future security, and environmental or housing conditions.

Although exploring the risk factors for autism with unknown causes is critical, the study has many limitations. For example, the study’s cross-sectional design introduces recall biases, as approximately 50% of mothers were unable to recall their dietary habits. Furthermore, the subjective report highlights the health effects of frequent exposure to seven daily life stressors during pregnancy. The web-based survey employs Delphi sampling techniques and excludes mothers with complex medical, psychological, or mental disorders, potentially influencing the results’ generalizability.

## 5 Conclusion

This study highlighted the maternal risk factors associated with autism spectrum disorders (ASD) in children. ASD is a diverse group of conditions characterized by some degree of difficulty with social interaction and communication.

Our study has shown a significant connection between gestational diabetes and autistic children, acknowledging the importance of interventions, screening tests, and lifestyle modifications during pregnancy. We also found a link between the severity of ASD and oxygen desaturation at birth. This highlights the importance of intervention in such cases to improve the child’s quality of life. Moreover, the mother’s stress and physical activity during household chores showed increased development of ASD in children, but a wider study is necessary to confirm the exact mechanism. Finally, the child’s age. The odds of having a more severe ASD increase with age.

## 6 Recommendation

After conducting this study, we concluded that understanding the relationship between the discussed factors and ASD development in children enables us to more effectively direct support services to meet the needs of these mothers and their children, enhancing their overall well-being.

- We recommend including social, psychological, work, and even financial support elements as essential components of the antenatal care program. Our findings reveal that over sixty percent of mothers with ASD faced daily challenges during pregnancy, which were related to the following: Factors include household chores, spouse conflict, time pressure, and financial concerns.
- Mass media health education campaigns aim to raise public awareness about the symptoms of ASD. In addition to educating mothers and fathers about sub categorical classification, management plans, prognosis, and ways to manage stigma-related issues and improve their life quality,
- To reduce the risk of ASD, the antenatal program should be comprehensive and include increased consumption of prenatal vitamins and supplements.
- These findings highlight the importance of monitoring and managing metabolic conditions, including gestational diabetes and infections, during pregnancy to mitigate potential risks to the child’s neurodevelopment.
- To strengthen the body of evidence, we recommend conducting similar studies on a random and representative sample in different settings, including mothers with complex medical, psychological, or mental disorders.
- Proper natal and postnatal care, including early and effective interventions for children with perinatal complications, has the potential to reduce ASD severity.

## Data Availability

The raw data supporting the conclusions of this article will be made available by the authors, without undue reservation.
