# Supplementary material for: Unveiling the influences of prenatal and maternal factors on the journey of an autistic child
Source: Front Psychiatry. 2024 Dec 20;15:1467821. doi: 10.3389/fpsyt.2024.1467821 (PMC11695324; doi:10.3389/fpsyt.2024.1467821)
Supplement: Supplementary file 1 [file Table1.docx]

**Table 1. Characteristics of included studies**

| **Study and year of publication** | **Study design** | **Country** | **Sample size** | **Treatment group** | **Control group** | **Age of participant** | **Protocol and follow-up** | **Outcome and measures** |
| --- | --- | --- | --- | --- | --- | --- | --- | --- |
| Ghanipour Badelbuu et al. 2019 (5) | Double-blinded randomized clinical trial | Iran | 90 | Aloe Vera (n=30) | -Routine treatment (hydrocortisone, clotrimazole, and zinc oxide) (n=30)  - Chamomile (n=30) | - 90.96 ± 140.20 days in Aloe Vera group  - 55.58 ± 146.73 days in Routine treatments  - 55.58 ± 146.73 days in Chamomile group | Three times a day. | Severity of Diaper Dermatitis (Size Scale by Dabirian et al. (6)) was determined on  the first, third, and sixth days of the study |
| Heidari et al. 2020 (7) | Double-blinded randomized clinical trial | Iran | 60 | Aloe Vera (n=30) | Routine combination treatment (hydrocortisone, clotrimazole, and zinc oxide) (n=30) | < 2 years | Three times a day. | Severity of Diaper Dermatitis (five-point  diaper rash instrument was used based on a study by  Al-Waili conducted in the United States (8)) was determined on  the first, third, and sixth days of the study |
| Amiri Farahani et al. 2013 (9) | Randomized clinical trial | Iran | 141 | Human breast milk (n=71) | Hydrocortisone 1% (n=70) | -4.6 ± 12.4 months in the breast milk group  -4.7 ± 11.2 months in the hydrocortisone group. | Twice a day for 7 days | Severity of Diaper Dermatitis (6-point scale) |
| Gozen et al. 2012 (10) | Randomised controlled prospective experimental study | Turkey | 63 | Human breast milk (n=30) | Barrier cream: 40% zinc oxide with cod liver  oil formulation (n = 33) | Mean 34.79 ± 2.86  weeks | Eight times a day for 5 days | Severity of Diaper Dermatitis (4-point scale (11)) |
| Adib-Hajbaghery et al. 2014 (a) (12) | Double-blinded randomized clinical trial | Iran | 60 | Calendula (n=30) | Bentonite (n=30) | -5.68 ± 0.84 months  in Calendula group  -6.88 ± 1.02 months in Bentonite group | Four times a day for 3 days | The effect of treatment (improvement or non-improvement) was documented based on the physician’s decision. |
| Adib-Hajbaghery et al. 2014 (b) (13) | A randomized, double blind, parallel controlled, non-inferiority trial | Iran | 60 | Calendula (n=30) | Shampoo-clay (n=30) | -6.88 ± 5.62 in Shampoo-clay  -5.68 ± 4.63 in Calendula group | Four times a day for 3 days | The effect of treatment (healed or not) was documented  based on the physician's decision. |
| Mahmoudi et al. 2015 (14) | Double-blinded randomized clinical trial | Iran | 100 | Calendula (n=50) | Bentonite (n=50) | -6.45±5.53 months in Calendula group --7.35±6.28 months in  Bentonite group | Four times a day for three days | The effect of treatment  (Improvement or non-improvement) was documented  based on the physician’s decision. |
| Panahi et al. 2012 (15) | Double-blinded randomized clinical trial | Iran | 66 | Calendula (n=34) | Aloe Vera (n=32) | < 3 years | Three times a day for 10 days | Severity of Diaper Dermatitis (5-point scale according to Davis et al. (16)) |
| Sharifi-Heris et al. 2018 (17kl7) | A triple-blind randomized clinical trial | Iran | 76 | Calendula (n=39) | Olive oil (n = 37) | -7.56±5.27 months in olive oil group  -7.2±4.3 months in Calendula group | Seven days | Severity of Diaper Dermatitis (6-point scale (18)) |
| Afshari et al. 2015 (19) | Double-blinded randomized clinical trial | Iran | 90 | Chamomile (n=45) | Calendula (n=45) | - 8. 8 ± 2.3 months in the Chamomile group  - 9.0 ±2.4 months in Calendula group | Four times a day for a week | Severity of Diaper Dermatitis (Diaper Rash five-point scale) |
| Ghanipour Badelbuu et al. 2019 (5) | Double-blinded randomized clinical trial | Iran | 90 | Chamomile (n=30) | -Routine treatment  (Hydrocortisone, clotrimazole, and zinc oxide) (n=30)  -Aloe Vera (n=30) | - 103.00 ± 146.73 days in Chamomile group  - 55.58 ± 146.73 days in control group  - 90.96 ± 140.20 days in Aloe Vera group | Three times a day. | Severity of Diaper Dermatitis (Size Scale by Dabirian et al. (6)) was determined on  the first, third, and sixth days of the study |
| Concannon et al. 2001 (11) | Double-blinded randomized placebo-controlled trial | Australia | 202 | Miconazole nitrate 0.25% (n= 101) | Ointment base (n=101) | 2-13 months | At each diaper change 7 days | Severity of Diaper Dermatitis (5-point scale) |
| Spraker et al. 2006 (20) | Double-blind, vehicle-controlled,  randomized, parallel-group, multicenter study | USA | 236 | Miconazole nitrate 0.25% (n=112) | Zinc oxide /petrolatum  vehicle (n=124) | - 7.67±4.87 in Miconazole nitrate 0.25% group  - 9.59±6.89 in Zinc oxide /petrolatum  Vehicle group | At each diaper change and after each bath for a total of 7 days | Severity of Diaper Dermatitis |
| Arad et al. 2009 (21) | Randomized clinical trial | Israel | 54 | Zinc oxide (n=18) | -Clobetasone butyrate (n=18)  -Eosin (n=18) | -7.3 ± 5.3 in Zinc oxide group  -11.2 ± 7.9 in Clobetasone butyrate group  -9.4 ± 7 in eosin group | -Zinc oxide: 6 times per day  -Clobetasone butyrate: 3 times per day  -Eosin: 6 times per day  For maximum 10 days | Severity of Diaper Dermatitis (5-point scale (16)) |
| Wananukul et al. 2006 (22) | A prospective, block randomized, investigator blinded study | Thailand | 46 | Zinc oxide and dexpanthenol (n=46) | Ointment base (liquid paraffin, bee wax) (n=46) | 1-22 months | 7 days | Severity of Diaper Dermatitis (6-point scale) |
| Dastgheib et al. 2017 (23) | A Single Blinded Non-  Randomized Controlled Trial | Iran | 58 | Hydrocortisone 1% (n=21) | Coriandrum sativum (n=37) | < 2 years | Twice a day for 10 days | Severity of Diaper Dermatitis |
| Keshavarz et al. 2016 (24) | A triple-blind, randomized trial, | Iran | 82 | Hydrocortisone 1% (n=41) | Henna (n=41) | -70.53 days in Henna group  -46.43 days in Hydrocortisone 1% group | Three times a day for 5  days. | Severity of Diaper Dermatitis (6-point scale) |

**Table 2. Sensitivity analysis for Calendula treatment.**

| **Study removed** | **Odds Ratio (95% CI)** | ***P*** |
| --- | --- | --- |
| **Calendula** | | |
| Adib-Hajbaghery et al. 2014 (a) | 0,168 (0,080- 0,354) | 0,000 |
| Adib-Hajbaghery et al. 2014 (b) | 0,168 (0,080- 0,354) | 0,000 |
| Mahmoudi et al. 2015 | 0,144 (0,059- 0,351) | 0,000 |
| Panahi et al. 2012 | 0,105 (0,053- 0,206) | 0,000 |
| Sharifi-Heris et al. 2018 | 0,121 (0,062- 0,239) | 0,000 |
