# Supplementary material for: Unveiling the influences of prenatal and maternal factors on the journey of an autistic child
Source: Front Psychiatry. 2024 Dec 20;15:1467821. doi: 10.3389/fpsyt.2024.1467821 (PMC11695324; doi:10.3389/fpsyt.2024.1467821)
Supplement: Supplementary file 2 [file DataSheet1.docx]

**Appendix**

| **A questionnaire to study autism factors during pregnancy** | **استبيان لدراسة عوامل التوحد أثناء فترة الحمل** |
| --- | --- |
| **Section 1 of 2:** | |
| Do you agree:   - Yes | هل أنت موافق:   - نعم |
| Mother's age (in years) during pregnancy | عمر الأم ( بالسنين ) أثناء فترة الحمل: |
| Father’s age (in years) during mother’s pregnancy | عمر الأب ( بالسنين )  أثناء فترة الحمل: |
| Place of residence during pregnancy:   - In the city - Outside the city | محل السكن اثناء فترة الحمل:   - في المدينة - خارج المدينة |
| Have you received any treatment to stimulate ovulation or insemination methods (intracytoplasmic injection - tube pregnancy)?   - Yes - No | هل تلقيت اي علاج لتنشيط التبويض او وسائل التقليح (حقن مجهري - حمل أنابيب)؟   - نعم - لا |
| Choose what you think applies to this pregnancy:   - I did not want this pregnancy - I wanted this pregnancy and it was planned - Artificial insemination - I did not know about my pregnancy until some time later - I became pregnant despite using contraception - Doctors warned me about pregnancy | اختر ما تراه ينطبق على هذا الحمل:   - لم أكن اريد هذا الحمل - أريد هذ الحمل ومخطط له - تلقيح صناعي - لم أكن اعلم بحملي الا بعد فترة - حملت علي الرغم من استخدامي وسيلة منع الحمل - حذرني الأطباء من الحمل |
| During pregnancy or during fertilization, was the father or mother exposed to any of the following factors (choose all that apply):   - None - Have been exposed to any type of radiation - You took any medications without a prescription - Other | في أثناء فترة الحمل أو أثناء التخصيب, هل تعرض (الأب او الأم) لأي من العوامل الآتية (اختر كل ما ينطبق:   - لا يوجد - تعرضت لأي نوع من الإشعاعات - تناولت أي عقاقيرعلاجية بدون وصفة طبية - أخرى |
| Select what applies:   \|  \| Both \| Father only \| Mother only \| Do not apply \| \| --- \| --- \| --- \| --- \| --- \| \| Ex-smoker \|  \|  \|  \|  \| \| Cigarette smoker \|  \|  \|  \|  \| \| Soft drinks \|  \|  \|  \|  \| \| Artificial sweetener (aspartame) \|  \|  \|  \|  \| | اختر ما ينطبق:   \|  \| كلاهما \| الاب فقط \| الام فقط \| لا ينطبق \| \| --- \| --- \| --- \| --- \| --- \| \| مدخن سابق \|  \|  \|  \|  \| \| مدخن سجائر \|  \|  \|  \|  \| \| مشروبات غازية \|  \|  \|  \|  \| \| سكر دايت (الاسبرتام) \|  \|  \|  \|  \| |
| Do you suffer from any chronic disease?   \|  \| Father \| Mother \| Both \| None \| \| --- \| --- \| --- \| --- \| --- \| \| Yes, a psychological or neurological illness \|  \|  \|  \|  \| \| Yes, a physical illness \|  \|  \|  \|  \| \| There are hereditary or familial cases of autism \|  \|  \|  \|  \| | هل تعاني من اي مرض مزمن؟   \|  \| الاب \| الام \| كلاهما \| لا يوجد \| \| --- \| --- \| --- \| --- \| --- \| \| نعم مرض عضوي نفسي او عصبي \|  \|  \|  \|  \| \| نعم مرض عضوي جسدي \|  \|  \|  \|  \| \| يوجد حالات وراثي او أسري لمرض التوحد \|  \|  \|  \|  \| |
| Before pregnancy, did you suffer from:   \|  \| No \| Yes \| I don’t know \| \| --- \| --- \| --- \| --- \| \| Cysts on the ovaries \|  \|  \|  \| \| Type 1 diabetes \|  \|  \|  \| \| Type 2 diabetes \|  \|  \|  \| \| Hypertension \|  \|  \|  \| \| High blood triglycerides \|  \|  \|  \| \| Asthma or difficulty breathing crises \|  \|  \|  \| | هل قبل الحمل كنت تعاني من:   \|  \| لا \| نعم \| لا أعرف \| \| --- \| --- \| --- \| --- \| \| تكيسات على المبايض \|  \|  \|  \| \| مرض السكري النوع الأول \|  \|  \|  \| \| مرض السكري النوع الثاني \|  \|  \|  \| \| ضغط الدم \|  \|  \|  \| \| ارتفاع الدهون الثلاثية في الدم \|  \|  \|  \| \| الربو او ازمات صعوبة في التنفس \|  \|  \|  \| |
| Were you exposed or injured during pregnancy:   \|  \| Yes \| No \| \| --- \| --- \| --- \| \| Eclampsia \|  \|  \| \| Diabetes during pregnancy \|  \|  \| \| To be infected with the new Corona virus \|  \|  \| \| Received the Corona vaccine \|  \|  \| \| Received other vaccines \|  \|  \| \| Have you ever had any seizures or convulsions? \|  \|  \| \| Took medication for epilepsy or seizures \|  \|  \| \| Took any medication for depression \|  \|  \| \| Antibiotics \|  \|  \| \| For alarming miscarriage or bleeding during pregnancy \|  \|  \| | هل تعرضت او اصبت أثناء الحمل:   \|  \| نعم \| لا \| \| --- \| --- \| --- \| \| تسمم الحمل \|  \|  \| \| سكرأثناء الحمل \|  \|  \| \| للأصابة بفيروس كرونا المستجد \|  \|  \| \| لتلقيت لقاح الكرونا \|  \|  \| \| تلقيت لقاحات أخرى \|  \|  \| \| عانيت من اي نوبات صرع او تشنجات \|  \|  \| \| تناولتي علاج للصرع او التشنجات \|  \|  \| \| تناولتي اي علاج للأكتئاب \|  \|  \| \| مضادات حيوية \|  \|  \| \| للآجهاض المنذر او نزيف اثناء الحمل \|  \|  \| |
| How many servings of cereals and bread did you eat daily during pregnancy? (A serving equals 1 slice of bread (25g) or 1/2 cup of cooked cereal or breakfast cereal or 4-6 medium-sized biscuits:   - Less than 6 servings - From 6 to 11 servings - More than 11 servings - I do not remember | كم عدد حصص الحبوب والخبز التي كنت تتناولها يوميًا اثناء الحمل؟ (الحصة تساوي شريحة خبز -25 جم- أو نصف كوب من الحبوب المطبوخة أو حبوب الإفطار أو 4-6 بسكويت متوسط ​​الحجم:   - أقل من ٦ حصص - من ٦ الي ١١ حصة - أكثر من ١١ حصة - لا أتذكر |
| How many servings of meat and legumes do you eat daily? (One serving is equivalent to 60-90 grams of red meat, chicken, fish, or half a cup of cooked legumes):   - Less than two servings - 2-3 servings - More than 3 servings - I never eat it - I'm a vegetarian - I do not remember | كم عدد حصص اللحوم والبقوليات التي تتناولها يوميًا؟ (الحصة تعادل 60-90 جم من اللحوم الحمراء أو الدجاج أو السمك أو نصف كوب من البقوليات المطهية):   - أقل من حصتين - ٢-٣ حصة - أكثر من 3 حصص - لا أتناوله ابدا - انا نباتي - لا أتذكر |
| How many servings of milk and dairy products do you eat daily? (A serving is equal to a cup of milk or yogurt - 240 ml - or 30 g of cheese):   - Less than 3 servings - From 3 to 5 servings - More than 5 servings - I do not remember | كم عدد حصص الحليب ومنتجات الألبان التي تتناولها يوميًا؟ (الحصة تساوي كوبًا من الحليب أو الزبادي - 240 مل - أو 30 جم من الجبن):   - أقل من ٣ حصص - من ٣ الي خمس حصص - اكثر من خمس حصص - لا أتذكر |
| How many servings of vegetables do you eat per day? (One serving equals a cup of vegetables, half a cup of juice, or half a cup of cooked vegetables):   - Less than 3 servings - From 3 to 5 servings - More than 5 servings - I do not remember | كم عدد حصص الخضار التي تتناولها في اليوم؟ (الحصة تساوي فنجان خضار أو نصف كوب عصير أو نصف كوب خضار مطبوخ)   - أقل من ٣ حصص - من ٣ الي خمس حصص - اكثر من خمس حصص - لا أتذكر |
| How many servings of fruit do you eat daily? (One serving equals 1 medium apple, orange, or banana, 1/2 cup - 120 ml - juice, or 1/2 cup dried fruit):   - Less than two servings - 2-3 servings - More than 3 servings - I do not remember | كم عدد حصص الفاكهة التي تتناولها يوميا؟ (الحصة تساوي تفاحة متوسطة أو برتقال أو موز أو نصف كوب - 120 مل - عصير أو نصف كوب من الفواكه المجففة)   - أقل من حصتين - ٢-٣ حصة - أكثر من 3 حصص - لا أتذكر |
| How many cups of water do you drink daily? (A cup is 240 ml):   - Less than 7 cups - 7-9 cups daily - More than 9 cups per day | كم كوب من الماء تشرب يوميا؟  ( الكوب 240 ملل):   - أقل من سبع اكواب - ٧ -٩ اكواب يوميا - آكثر من تسع أكواب يوميا |
| Average physical activity during pregnancy:   - Only basic needs - Routine household activities - I practice walking (half an hour, three to four times a week) or moderate physical activity - I do more physical activity (walking an hour and a half a week) or vigorous physical activity | متوسط النشاط البدني أثناء فترة الحمل:   - فقط الاحتياجات الاساسية - الأنشطة المنزلية الروتينية - أقوم بممارسة المشي (نصف ساعة من ثلاث لاربع مرات في الاسبوع) او نشاط بدني متوسط - أقوم بنشاط بدني أكثر (مشي ساعة ونصف بالاسبوع) او نشاط بدني شديد |
| Have you measured your testosterone level during pregnancy?   - No - Yes (it was normal) - Yes (it was abnormal) | هل قمتي بقياس نسبة هرمون التيستستيرون اثناء الحمل   - لا - نعم (كان طبيعي) - نعم (كان غير طبيعي) |
| Choose what you think applies during pregnancy:   \|  \| No \| Yes \| To some extent \| \| --- \| --- \| --- \| --- \| \| Suffered from anemia \|  \|  \|  \| \| Suffered from frequent infections \|  \|  \|  \| \| Taking nutritional supplements (calcium and iron during pregnancy) \|  \|  \|  \| \| Periodic follow-up with a specialist doctor \|  \|  \|  \| \| Did you take folic acid before pregnancy/or during the first months of pregnancy? \|  \|  \|  \| \| Fever, joint pain, headache, or flu-like symptoms \|  \|  \|  \| \| You have experienced excessive vomiting (severe morning sickness) \|  \|  \|  \| \| Sleep disturbances and difficulties (decreased number of hours of sleep) \|  \|  \|  \| \| You were exposed to air pollution, such as high carbon levels \|  \|  \|  \| \| You were exposed to high doses of lead or arsenic \|  \|  \|  \| | اختر ما تراه ينطبق أثناء فترة الحمل:   \|  \| لا \| نعم \| الى حد ما \| \| --- \| --- \| --- \| --- \| \| عانيت من انيميا \|  \|  \|  \| \| عانيت من التهابات متكررة \|  \|  \|  \| \| تناول المكملات الغذائية (الكالسيوم والحديد اثناء الحمل) \|  \|  \|  \| \| المتابعة الدورية عند الطبيب المختص \|  \|  \|  \| \| هل تناولت الفوليك اسيد قبل الحمل / او في الشهور الأولى للحمل \|  \|  \|  \| \| من الحمى أو آلام المفاصل أو الصداع أو أعراض تشبه أعراض الأنفلونزا \|  \|  \|  \| \| عانيت من القيء المفرط (غثيان الصباح الشديد) \|  \|  \|  \| \| اضطرابات وصعوبات في النوم (انخفاض عدد ساعات النوم) \|  \|  \|  \| \| تعرضتي الى تلوث جوي مثل ارتفاع نسبة الكربون \|  \|  \|  \| \| تعرضتي الى جرعات عالية من مادة الرصاص او الارسينك \|  \|  \|  \| |
| Did you use any medication during pregnancy, and if so, what was it? | هل استخدمتي أي دواء خلال فترة الحمل، وإذا كان الأمر كذلك، فما هي؟ |
| During pregnancy, did any of the following factors have a bad or worsening effect on health?   \|  \| 0 None at all \| 1  It exists but has no effect \| 2 Negative effect \| 3  Very negative effect \| \| --- \| --- \| --- \| --- \| --- \| \| Domestic troubles \|  \|  \|  \|  \| \| \| The husband \|  \|  \|  \|  \| \| \| Time pressure of too many things to do \|  \|  \|  \|  \| \| \| Internal fears (fears of being alone, fear of confrontation) \|  \|  \|  \|  \| \| \| Environmental factors (neighbours, crime, deterioration \|  \|  \|  \|  \| \| \| Financial responsibility \|  \|  \|  \|  \| \| \| Work-related factors (problems with colleagues or dissatisfaction with the job) \|  \|  \|  \|  \| \| \| Future security (job - inability to work) \|  \|  \|  \|  \| \| | أثناء فترة الحمل, هل اي من العوامل الأتية كان له تأثير سيء على الصحة او ادى الى تفاقمها؟   \|  \| 0  لا يوجد اصلا \| 1  يوجد ولكن ليس له تاثير \| 2  تأثير سلبي \| 3  تاثير سلبي جدا \| \| --- \| --- \| --- \| --- \| --- \| \| المتاعب المنزلية \|  \|  \|  \|  \| \| الزوج \|  \|  \|  \|  \| \| ضغط الوقت الكثير من الأشياء التي يجب القيام بها \|  \|  \|  \|  \| \| المخاوف الداخلية (المخاوف من أن تكون وحيدا، والخوف من المواجهة) \|  \|  \|  \|  \| \| عوامل بيئة ( الجيران او الجريمة ، تدهور \|  \|  \|  \|  \| \| المسؤولية المالية \|  \|  \|  \|  \| \| عوامل تتعلق بالعمل (مشاكل مع الزملاء او عدم الرضا عن الوظيفة) \|  \|  \|  \|  \| \| الأمن المستقبلي (الوظيفة - عدم القدرة على العمل) \|  \|  \|  \|  \| |
| **Section 2 of 2:** | |
| Information about the birth of the child | معلومات عن ولادة الطفل |
| The baby was born:   - Premature birth - At his appointed time - After the specified time | كانت ولادة الطفل:   - ولادة مبكرة - في الوقت المحدد له - ولادة بعد الوقت المحدد |
| In what week was the baby born (in weeks or months)? | في اي اسبوع كانت ولادة الطفل (بالاسبوع او الشهور)؟ |
| Place of birth:   - At home - In a private hospital - In a public hospital - In a clinic | مكان الولادة:   - في البيت - في مستشفى خاص - في مستشفى حكومي - في عيادة |
| Child's current age: | عمر الطفل الحالي: |
| Type of birth:   - Natural delivery without complications - Natural delivery - Caesarean delivery - Caesarean section (with some complications or difficulties) | نوع الولادة:   - ولادة طبيعية بدون مضاعفات - ولادة طبيعية - ولادة قيصرية - ولادة قيصرية (مع وجود بعض المضاعفات او الصعوبات) |
| Were you anesthetized during childbirth?   - No - Yes, local anesthesia - Yes, spinal anesthesia - Yes, general anesthesia | هل تم تخديرك أثناء الولادة؟   - لا - نعم, تخدير موضعي - نعم, تخدير نصفي - نعم, تخدير كلي |
| Choose what you think applies to the child at birth:   \|  \| Less than normal \| Normal \| More than normal \| I don’t know \| \| --- \| --- \| --- \| --- \| --- \| \| Weight \|  \|  \|  \|  \| \| Height \|  \|  \|  \|  \| | اختر ما تراه ينطبق على الطفل عند الولادة:   \|  \| اقل من الطبيعي \| طبيعي \| أكبر من الطبيعي \| لاأعرف \| \| --- \| --- \| --- \| --- \| --- \| \| الوزن \|  \|  \|  \|  \| \| الطول \|  \|  \|  \|  \| |
| Choose what applies to the child:   \|  \| No \| Yes \| \| --- \| --- \| --- \| \| Has the child entered nursery \|  \|  \| \| Infections \|  \|  \| \| Epileptic seizures \|  \|  \| \| Low oxygen level \|  \|  \| | أختر ما ينطبق على الطفل:   \|  \| لا \| نعم \| \| --- \| --- \| --- \| \| هل دخل الطفل حضانة \|  \|  \| \| التهابات \|  \|  \| \| نوبات صرع \|  \|  \| \| نقص نسبة الأوكسجين \|  \|  \| |
| Who was the first to diagnose him?   - The family - The doctor | من اول من شخصه؟   - الأسرة - الطبيب |
| How was the diagnosis made?   - By symptoms only - According to diagnostic criteria (radiography and laboratory tests) - According to diagnostic criteria (with a multidisciplinary medical team) - Other | كيف تم التشخيص؟   - بالأعراض فقط - طبقا لمعايير التشخيص (بالأشعة والتحاليل المخبرية) - طبقا لمعايير التشخيص (بفريق طبي متعدد التخصصات) - أخرى |
| What is the degree or severity of your child's autism?   - Not specified - Other/or autism spectrum - Simple autism - Moderate autism - Severe autism - I don't know | ما هي درجة أو شدة الاصابة لدى طفلك؟   - لم يتم تحديدها - اخرى / او طيف التوحد - توحد بسيط - توحد متوسط - توحد شديد - لا أعرف |
| **Thank you for your cooperation with us** | **شكرا لتعاونكم معنا** |
